# The Value of Statistics in Cataphoresis

**Published:** 1897-01

**Authors:** L. E. Custer

**Affiliations:** Dayton, Ohio


### THE VALUE OF STATISTICS IN CATAPHORESIS.

BY L. E. CUSTER, B.S., D.D.S., DAYTON, OHIO.

THE December, 1896, number of the INTERNATIONAL DENTAL JOURNAL contains a record of forty cataphoric operations by Dr. Louis Jack. Considerable time and care must have been given to obtain the data recorded, and while the effort is certainly commendable, we do not see that the data are of much practical benefit to other operators, or of much value in establishing rules and tables for reference for his own or the use of others operating even the same appliance, unless he can convert the number of cells and the contact points of the rheostat into volts.

The variations both in the manner and means of applying the current and in the cavity are of such wide range that it will always be difficult to obtain accurate data in these operations, and yet if we are to make a success of cataphoresis we must make these records and learn what we can from them.

By the title of the paper Dr. Jack does not intend that they should be more than notes on cataphoresis, and yet these notes could be made of great value if, instead of giving the number of cells and the pins of the rheostat, he had given the pressure in volts as measured on a standard volt-meter.

The voltage of the different cells on the market is not the same, nor does the voltage of a single cell remain the same during all its life. By the construction of a cell and its elements there may be a wide range in the voltage. A Smee or a Walker cell will produce an electrometric force of but half a volt, while a chromic acid, or a Fuller cell, would give two volts or four times the former. This shows that it is important to know the kind of cell being used.

Again, the chemical action going on in any given cell is not the same at all times. In some cells there is a steady voltage for a time, and then a gradual decline. This is characteristic of open circuit cells. In a dry cell there appears to be a "warming up" of the chemical process, and with it a rise in the voltage followed by a drop. At the close of the life of any cell there is always a lower voltage than when the cell was new.

The rheostat used for controlling the current is also liable to variation. This is especially true of water and graphite instruments for this purpose. Water becomes a better conductor as it heats, while all metals act in just the reverse. This property of metals, except in the case of iron, is scarcely appreciable, but with water it is somewhat marked. The form of graphite rheostat, in which this material is contained in a powdered form in a flexible receptacle is even more uncertain than water, and those rheostats which are made of a thin coating of graphite upon slate or the like are not to be used at all unless a volt-meter is used with them.

Then, again, the conditions which pertain to the patient and the method of applying the current are important factors in securing reliable data. It was shown in a paper read before the March meeting of the Mississippi Valley Dental Society that the enamel is not a conductor of electricity; that it covers the dentine as an electrical insulator, about equal to so much porcelain. The dentine is a conductor only by virtue of the moisture contained in its tubuli, and anæsthesia is produced only in the fibrils contained in those tubuli whose mouths open into the cavity. The fibrils in those tubuli covered by enamel only become anæsthetized when the cocaine has reached the pulp through the exposed tubuli. We might say that, except where there is a very little penetration of the current laterally at the interzonal layer, unexposed dentine can only be anæsthetized reflexly. To do this the cocaine must be carried entirely through the dentine and the coronal portion of the pulp infiltrated.

Now, since the electric current, in flowing from the positive to the negative through the patient, flows through what we might call a constriction equal to the area of exposed dentine, and since no more can flow under a given pressure in volts than can pass this point, it is evident that the exposure of dentine and the distance to the pulp alone determines the current in ampères which will flow. Of course there is resistance between the pulp and the negative electrode on the cheek or hand, but that is so very small as compared to that offered by the dentine as to be negligible. A small cavity with a small area of dentine exposed under eight volts would allow, say, one-tenth milliampère to flow, while a large cavity under the same pressure would allow perhaps five-tenths milliampère. There is a distinct proportion between the area of exposed dentine and ampères under the same pressure in persons of about the same age.

The distance from the pulp—that is whether the cavity is shallow or deep—is a feature which relates more especially to the volts that may be attained than to the ampères. It will require a higher voltage in a superficial cavity than in a deep one, because the resistance is greater. This, at the same time, means that with a high voltage in a superficial cavity no more is accomplished than with a low voltage in a deep cavity, and it accounts for the length of time, and often the high voltage, required in this class of cavities.

That condition of dentine which has been long exposed to abrasion, and is without sensation, is much like enamel electrically. The tubuli have been filled in with lime salts, and it is almost a non-conductor. It is only when the deeper and sensitive layer is reached that we have the principal condition for cataphoresis,—namely, conductivity. Dentine of this character will offer greater resistance than freshly exposed dentine of young persons, and this must be borne in mind in the measurement of current.

The negative electrode should always be placed at the same part of the body, because, as has been shown by Dr. Price, there is a difference of from three thousand to five thousand ohms resistance between the cathode upon the cheek and upon the hand. It is desirable to keep the voltage down, and consequently the most desirable position for this electrode is upon the cheek.

There are so many variable conditions in the operation of cataphoresis that I do not think it is possible to previously estimate, with accuracy, the number of volts and ampères that a given case under operation will require, and yet by the tabulation of our cases, just as Dr. Jack has done, except that standard measuring instruments be used, I believe we will, after a little experience, be able thereby to tell just when we have gone far enough with the current. We will learn just what the requirements of each case will be, and can tell how nearly we are filling these requirements.

